# Physical Activity Differentially Affects the Cecal Microbiota of Ovariectomized Female Rats Selectively Bred for High and Low Aerobic Capacity

**DOI:** 10.1371/journal.pone.0136150

**Published:** 2015-08-24

**Authors:** Tzu-Wen Liu, Young-Min Park, Hannah D. Holscher, Jaume Padilla, Rebecca J. Scroggins, Rebecca Welly, Steven L. Britton, Lauren G. Koch, Victoria J. Vieira-Potter, Kelly S. Swanson

**Affiliations:** 1 Division of Nutritional Sciences, University of Illinois at Urbana-Champaign, Urbana, Illinois, United States of America; 2 Department of Animal Sciences, University of Illinois at Urbana-Champaign, Urbana, Illinois, United States of America; 3 Department of Nutrition and Exercise Physiology, University of Missouri, Columbia, Missouri, United States of America; 4 Dalton Cardiovascular Research Center, University of Missouri, Columbia, Missouri, United States of America; 5 Department of Child Health, University of Missouri, Columbia, Missouri, United States of America; 6 Department of Anesthesiology, University of Michigan, Ann Arbor, Michigan, United States of America; 7 Department of Molecular & Integrative Physiology, University of Michigan, Ann Arbor, Michigan, United States of America; University of Palermo, ITALY

## Abstract

The gut microbiota is considered a relevant factor in obesity and associated metabolic diseases, for which postmenopausal women are particularly at risk. Increasing physical activity has been recognized as an efficacious approach to prevent or treat obesity, yet the impact of physical activity on the microbiota remains under-investigated. We examined the impacts of voluntary exercise on host metabolism and gut microbiota in ovariectomized (OVX) high capacity (HCR) and low capacity running (LCR) rats. HCR and LCR rats (age = 27wk) were OVX and fed a high-fat diet (45% kcal fat) *ad libitum* and housed in cages equipped with (exercise, EX) or without (sedentary, SED) running wheels for 11wk (n = 7-8/group). We hypothesized that increased physical activity would hinder weight gain, increase metabolic health and shift the microbiota of LCR rats, resulting in populations more similar to that of HCR rats. Animals were compared for characteristic metabolic parameters including body composition, lipid profile and energy expenditure; whereas cecal digesta were collected for DNA extraction. 16S rRNA gene-based amplicon Illumina MiSeq sequencing was performed, followed by analysis using QIIME 1.8.0 to assess cecal microbiota. Voluntary exercise decreased body and fat mass, and normalized fasting NEFA concentrations of LCR rats, despite only running one-third the distance of HCR rats. Exercise, however, increased food intake, weight gain and fat mass of HCR rats. Exercise clustered the gut microbial community of LCR rats, which separated them from the other groups. Assessments of specific taxa revealed significant (*p*<0.05) line by exercise interactions including shifts in the abundances of *Firmicutes*, *Proteobacteria*, and *Cyanobacteria*. Relative abundance of *Christensenellaceae* family was higher (*p* = 0.026) in HCR than LCR rats, and positively correlated (*p*<0.05) with food intake, body weight and running distance. These findings demonstrate that exercise differentially impacts host metabolism and gut microbial communities of female HCR and LCR rats without ovarian function.

## Introduction

Recent studies have suggested that the gut microbiome plays a crucial role in the development of obesity and metabolic diseases. Profound changes in the gut bacterial phyla between lean and obese individuals have been revealed [1, 2]. The ratio of the two predominant bacterial phyla, *Bacteroidetes* and *Firmicutes*, has been reported to be altered in leptin-deficient *ob/ob* mice [3] and in obese human subjects [4]. Obesity has also been induced by transferring gut microbes from obese mice to gnotobiotic mice [3]. In that study, gnotobiotic mice receiving gut bacteria from obese mice gained more weight than those receiving gut bacteria from lean mice, without differences in food intake [3]. These results suggest that the gut microbiota affect the efficiency of energy harvest from the indigestible components of the diet and alter how the energy is used, influencing host energy balance and metabolism. Given the complexity of the gut microbiota, analyses must move beyond the mere comparison of the *Bacteroidetes*: *Firmicutes* ratio. More in-depth studies are needed to elucidate the physiological changes associated with specific populations of gut microbiota.

In the United States, 35% of adults and 17% of adolescents are obese [5, 6]. While the overall obesity prevalence in adolescents and adults has remained the same in the past decade, postmenopausal women represent one population whose obesity rates are increasing [7]. Menopause in humans and ovariectomy (OVX) in rodents is associated with significant increase in adiposity, reduced physical activity and aerobic fitness, and onset of metabolic disease. In contrast, intact female mice are protected against weight gain, and remained more glucose tolerant than weight-matched male mice after onset of obesity [8, 9].

Lifestyle modifications, including dietary and physical activity interventions are currently the main clinical approaches to prevent or treat obesity. Unlike other models of obesity where increased energy intake is a major causative factor, obesity induced by ovarian hormone loss is more related to reduced physical activity and not increased energy intake [10]. Thus, metabolic dysfunction associated with ovarian hormone loss is largely attributable to reduce physical activity, and the resulting lower aerobic fitness. Indeed, increasing physical activity and increasing cardiorespiratory fitness both independently improve metabolic dysfunction in models of ovarian hormone loss. Unfortunately, the unique mechanisms underlying these protective effects are not known. Regular exercise improves aerobic capacity, increases basal metabolic rate, and promotes many health-related benefits, whereas physical inactivity leads to low aerobic fitness that is strongly associated with early mortality, and incidence of type 2 diabetes (T2D), cardiovascular disease (CVD), and non-alcoholic fatty liver disease (NAFLD) [11, 12]. Completely unknown is how regular exercise and/or aerobic fitness impacts gut microbiota in the setting of OVX. Because up to 70% of the variation in the intrinsic aerobic capacity is believed to come from genetic background [13], we have taken advantage of a high capacity running (HCR) and low capacity running (LCR) rat model to investigate how intrinsic fitness affects the impact of voluntary exercise on gut microbiota following OVX.

Over several generations of selective breeding, a polygenic HCR/LCR rat model based on the ability of endurance running was developed [14, 15]. HCR and LCR have distinctly different intrinsic aerobic capacities and risk factors for various diseases [16]. LCR rats are heavier, have higher adiposity, and score higher on cardiovascular risk factors, metabolic syndrome and NAFLD risk factors compared to the HCR rats [17, 18]. HCR rats are protected against HFD-induced insulin resistance, have higher hepatic mitochondrial oxidative capacity, and a longer life span [14, 18]. We recently demonstrated that, compared to LCR, HCR rats are also protected against energy balance dysregulation and metabolic dysfunction following OVX [19]. Because bacteria may affect energy harvest and weight gain, we sought to assess whether gut bacteria were altered in an ovariectomized model that, similar to post-menopausal women, exhibits greater adiposity. Further, by using rats bred for high- and low- intrinsic aerobic capacity, we aimed to (1) identify differences imparted by intrinsic fitness on gut microbial communities; (2) determine the effect of exercise on gut microbial communities; and (3) determine if the effect of exercise on gut microbial communities differs based on intrinsic fitness level. Given the contrasting aerobic capacities even in a sedentary condition, this model is a robust tool to investigate the connection between the gut microbiota and intrinsic aerobic fitness. We hypothesized that increased physical activity would decrease weight gain, improve metabolic health and shift the microbiota of LCR rats, resulting in populations more similar to that of HCR rats.

## Materials and Methods

### Animals and Diets

All animal procedures were approved by the Institutional Animal Care and Use Committee at the University of Missouri, Columbia. The HCR/LCR rat model was established by Drs. Britton and Koch as previously described [17]. Briefly, these rats were selectively bred based on their endurance running capacity, which was determined by running time to exhaustion on a treadmill test. Fifteen female HCR rats and fifteen female LCR rats arrived at the University of Missouri at 19 wk of age (generation 33) and were single-housed under controlled humidity and temperature with a 12-h light/ 12-h dark cycle. All rats were fed regular rodent chow diet (LabDiet 5001, Purina) until group randomization at the beginning of the intervention. Food and water were always provided *ad libitum*.

### Intervention

At 26 wk of age, rats from each line (i.e., HCR, and LCR) were randomized to cages equipped with voluntary running wheels (11cm wide and 35cm in diameter; EX group) or remained in cages without running wheels to serve as sedentary controls (SED group) creating four groups as a 2x2 factorial design: 1) HCR-EX (n = 8), 2) HCR-SED (n = 7), 3) LCR-EX (n = 8), 4) LCR-SED (n = 7). Voluntary exercise was chosen for our study design due to the nature of HCR and LCR rats in terms of their distinct aerobic fitness capacity. Controlled bouts of exercise would potentially increase stress in LCR rats if amount of exercise chosen exceeded the capacity that LCR rats could handle, and stress related hormones are known to affect gut microbial communities [20]. Therefore, we chose to provide voluntary wheel running and recorded the running distance in the current study, in which we believe to be well translated to the human population. Running wheels were connected to an electronic bicycle computer monitoring system (Sigma Sport BC 800 bicycle computer, Cherry Creek Cyclery, Foster Fall, VA) to measure running distance. All rats were ovariectomized (OVX) at time of group randomization, allowed one week recovery, then placed into their prospective cages, and fed a 45% kcal fat diet (HFD) (D12451, Research Diets, New Brunswick, NJ) for the remainder of the study (11 wk). Body weight (BW), food intake, and running distance were measured weekly. Feed efficiency was calculated by dividing amount of energy consumed by each animal by the BW gain. Average feed efficiency (kcal consumed/g BW gain) reported in Table 1 represented the mean throughout the 11-wk period. Energy expenditure was measured using indirect calorimetry over a 3-d period at 36 wk of age. Although those data were not used to calculate efficiency, it provided an estimate on basal metabolic differences that may have, in part, impacted efficiency.

**Table 1:** Body weight, food intake and daily wheel running distance^1^.

	HCR		LCR		*p* values		
	EX	SED	EX	SED	Line	Trt	Line*Trt
Initial body weight (g)	231.3 ± 7.3	240.7 ± 7.8	271.2 ± 7.3	275.9 ± 7.8	< .0001	0.3595	0.7604
Final body weight (g)	330.0 ± 9.0	304.9 ± 9.6	327.5 ± 9.0	360.7 ± 9.6	0.0083	0.6685	0.0043
Body weight gain^2^ (g)	98.8 ± 7.3	64.2 ± 7.8	56.3 ± 7.3	84.8 ± 7.8	0.1616	0.6917	0.0003
Food intake (kcal/d)	88.7 ± 3.9	57.8 ± 4.1	62.4 ± 3.9	60.7 ± 4.1	0.0072	0.0004	0.0011
Feed efficiency (kcal consumed/g BW^3^ gain)	8.6 ± 2.8	13.2 ± 3.0	19.5 ± 2.8	9.4 ± 3.0	0.2440	0.3556	0.0184
Running distance (km/d)	5.0 ± 0.8	N/A	1.5 ± 0.3	N/A	0.0017	N/A	N/A
Energy expenditure^4^ (kcal/d)	50.8 ± 1.6	45.2 ± 1.7	43.9 ± 1.6	42.7 ± 1.7	0.0099	0.0514	0.1985

^1^n = 7-8/group, values are means ± SEM.

^2^Body weight, food intake and running distance were measured weekly. Feed efficiency was calculated by dividing amount of energy consumed by each animal by the BW gain. These data represent the mean throughout the 11-wk period.

^3^BW = body weight; HCR = high capacity runner rats; LCR = low capacity runner rats; EX = voluntary exercise; SED = sedentary.

^4^Energy expenditure was measured using indirect calorimetry over a 3-d period at 36 wk of age.

### Ovariectomy Surgeries

Surgical procedures were followed as previously described[21]. Briefly, rats were anesthetized using isofluorane during surgery. A one-inch incision was made at the midline of the dorsal surface, followed by two bilateral cuts through the muscle layer to expose the ovaries. For OVX, the whole ovary, including the ovarian bursa and part of the oviduct, were removed. The incision was closed using wound clips. Rats were administered Banamine (NSAID, 1.0 mg/kg) after the surgery to help relieve pain. OVX surgery effectiveness was determined at the conclusion of the study via verification of significant uterine atrophy.

### Body Composition and Blood Parameters

As 38 wk of age, body composition was determined by using a Hologic QDR- 1000 dual-energy X-ray absorptiometry (DXA) instrument for rodents. Rats were anesthetized using pentobarbital (30-40mg/kg) after an 8-h fast, followed by cardiac puncture for blood collection. Following euthanization, subcutaneous, perigonadal, retroperitoneal, and omental fat pads were dissected and weighed. Serum, plasma, and cecal digesta were collected and stored at -80°C until further analysis. The analyses of glucose, cholesterol, triglycerides, and non-esterified fatty acids (NEFA) were performed by a commercial laboratory (Comparative Clinical Pathology Services, Columbia, MO) using an Olympus AU680 automated chemistry analyzer (Beckman-Coulter, Brea, CA) and commercially available assays, according to the manufacturer’s guidelines. Plasma insulin concentrations were determined using rat-specific ELISA kit per manufacturer’s instructions (Alpco Diagnostics, Salem, NH). Cecal digesta dry matter and crude protein concentrations were measured using procedures by the Association of Official Analytical Chemists (dry matter: method 934.01; crude protein: method 992.15)[22].

### Energy Expenditure

A metabolic monitoring system (Promethion, Sable Systems Int., Las Vegas, NV) measuring oxygen consumption, carbon dioxide production, and a multi-dimensional beam break system was employed to assess the total and resting energy expenditure over a 72-hr period, 8 wk following OVX. Animals were singly housed in the metabolic chamber system and allowed to acclimate to the chamber environment for one day before data collection. Data were analyzed as daily and cycle averages (i.e., 24-hr as well as individual 12-hr light and dark cycle averages) and were calculated per animal; these daily averages were then used to calculate group means.

### Cecal Digesta DNA Extraction, Amplification, Sequencing and Bioinformatics

Total DNA from cecal digesta samples was extracted using Mo-Bio PowerSoil kits (MO BIO Laboratories, Inc., Carlsbad, CA)[23]. Concentration of extracted DNA was quantified using a Qubit 2.0 Fluorometer (Life technologies, Grand Island, NY). 16S rRNA gene amplicons of 250 bp were generated from the V4 region as described by Caporaso et al. [24]. Amplicons were purified using Agencourt AMPure XP DNA purification beads (Beckman Coulter Genomics GmbH) to remove fragments less than 300 bp. Quality of the amplicons was assessed using electrophoresis with precast agarose E-gels (Invitrogen, Grand Island, NY) and by using a 2100 Bioanalyzer (Agilent Technologies, Santa Clara, CA). The Qiagen gel purification kit was then used to remove the high molecular weight genomic DNA before generating a DNA pool by combining equimolar amounts of the amplicons from each sample. Illumina sequencing was performed on a MiSeq2000 using v2 reagents (Illumina Inc., San Diego, CA) at the W. M. Keck Center for Biotechnology at the University of Illinois. QIIME 1.8.0 was used to process the resulting sequence data [25]. Briefly, high quality (quality value > 25) sequence data derived from the sequencing process were demultiplexed and quality filtered using split_libraries_fastq.py default parameters. Sequences were then clustered into operational taxonomic units (OTU) using closed-reference OTU picking against the Greengenes 13_8 reference OTU database with a 97% similarity threshold [26]. An even sampling depth of 4063 sequences per sample was used for assessing alpha- and beta-diversity measures.

### Statistical Analyses

All data were analyzed using SAS 9.2 (SAS Institute, Cary, NC) with Mixed Models procedure. When appropriate, post hoc Tukey’s tests were performed to determine the differences among groups (i.e. HCR-EX, HCR-SED, LCR-EX, and LCR-SED groups). Data normality was checked using the UNIVARIATE procedure and Shapiro-Wilk statistic. Correlations between the relative microbial abundances and physiological profile were assessed using Pearson correlation coefficient. Data are reported as means ± SEM with statistical significant set as *p*<0.05 and *p* <0.10 considered as trends.

## Results

### Animal Characteristics and Running Capacity

The initial mean BW of LCR rats was approximately 16% higher (*p*<0.05) than that of HCR rats, with no differences observed between the EX and SED groups within each line (Table 1). A line (i.e., HCR vs. LCR) by treatment (i.e., EX vs. SED) interaction (*p*<0.05) was observed for final BW, such that exercise reduced BW in LCR rats (*p*<0.05), but increased BW (*p*<0.05) in HCR rats. The HCR-EX group also consumed more energy (*p*<0.05) than the other 3 groups, whereas exercise did not affect food intake of LCR rats. A line by treatment interaction was also noted with feed efficiency, represented as calories consumed:BW gain, over the 11-wk study. Increased (*p*<0.05) feed efficiency was observed in the HCR-EX group, but decreased (*p*<0.05) in LCR-EX rats. That is, it required nearly twice as many calories for LCR-EX rats to gain 1 gram of BW compared to LCR-SED rats. HCR-EX rats also ran >3-times longer distance (*p*<0.05) than LCR-EX rats (5.0 ± 0.8 vs. 1.5 ± 0.3 km/d, Table 1). Finally, HCR rats had greater (*p*<0.05) daily energy expenditure than LCR rats (Table 1).

### Body Composition, Blood Parameters, and Cecal Protein

Similar to the BW gain differences, line by treatment interactions (*p*<0.05) were observed for total fat mass and percent body fat, with exercise significantly reducing adiposity in LCR rats, but increasing adiposity in HCR rats (Table 2). Moreover, at the completion of the study, LCR-EX rats had less than half of the body fat mass of LCR-SED rats. Lean body mass (LBM) tended to increase (*p* = 0.067) with exercise in both strains, although a significant line by treatment interaction (*p*<0.05) was observed in percent LBM, such that exercise increased percent LBM only in the LCR rats. Fasting glucose did not differ between HCR and LCR or between EX and SED, but LCR rats tended to have 34% higher (*p* = 0.06) fasting insulin compared to HCR rats (Table 2). Total, LDL, and HDL cholesterol were all higher (*p*<0.05) in the LCR rats compared to HCR rats. Moreover, significant line by treatment interactions (*p*<0.05) were observed for fasting triglyceride and NEFA concentrations, and for subcutaneous, perigonadal, retroperitoneal, and omental fat pad weights, with exercise decreasing these outcomes in LCR rats but increasing them in HCR rats. Cecal digesta dry matter did not differ between strains or treatment. A significant line by treatment interaction (*p*<0.05) was observed in cecal digesta crude protein concentration, however, with exercise decreasing the cecal digesta crude protein in the LCR rats, but slightly increasing it in HCR rats (Table 2).

**Table 2:** Body composition and metabolic parameters^1^.

	HCR		LCR		*p* values		
	EX	SED	EX	SED	Line	Trt	Line*Trt
Body fat mass^2^ (g)	59.06 ± 7.90	40.43 ± 8.45	54.00 ± 7.90	111.49 ± 8.45	0.0004	0.0252	< .0001
Lean body mass^2^ (g)	262.27 ± 7.14	256.94 ± 7.63	266.36 ± 7.14	243.43 ± 7.63	0.5292	0.0670	0.2442
Percent body fat mass (%)	17.53 ± 2.13	13.07 ± 2.28	15.99 ± 2.13	30.39 ± 2.28	0.0014	0.0330	0.0002
Percent lean body mass (%)	79.40 ± 1.62	83.82 ± 1.73	80.80 ± 1.62	66.81 ± 1.73	< .0001	0.0084	< .0001
Fasting glucose (mg/dL)	166.13 ± 5.50	153.86 ± 5.88	152.00 ± 5.88	160.14 ± 5.88	0.5042	0.7244	0.0899
Fasting insulin (ng/mL)	2.96 ± 0.62	2.02 ± 0.67	3.48 ± 0.67	4.05 ± 0.67	0.0632	0.7786	0.2596
HOMA-IR^3^	35.77 ± 7.90	22.85 ± 8.45	38.50 ± 8.45	48.09 ± 8.45	0.1050	0.8429	0.1879
Total cholesterol (mg/dL)	77.63 ± 5.66	71.14 ± 6.05	88.57 ± 6.05	92.93 ± 6.05	0.0109	0.8598	0.3713
LDL cholesterol (mg/dL)	42.08 ± 3.74	40.17 ± 4.00	51.80 ± 4.00	52.77 ± 4.00	0.0090	0.9068	0.7182
HDL cholesterol (mg/dL)	27.50 ± 1.42	24.43 ± 1.52	29.86 ± 1.52	30.36 ± 1.52	0.0105	0.3986	0.2442
Triglycerides (mg/dL)	40.25 ± 5.23	32.71 ± 5.59	34.57 ± 5.59	49.00 ± 5.59	0.3442	0.5367	0.0569
Fasting NEFA (mmol/L)	0.46 ± 0.06	0.31 ± 0.06	0.27 ± 0.06	0.41 ± 0.06	0.4899	0.9225	0.0334
Subcutaneous fat pad (g)	1.80 ± 0.33	1.52 ± 0.35	2.43 ± 0.33	4.01 ± 0.38	0.0001	0.0706	0.0118
Perigonadal fat pad (g)	9.72 ± 1.42	6.72 ± 1.52	8.62 ± 1.42	14.12 ± 1.52	0.0415	0.4035	0.0075
Retroperitoneal fat pad (g)	2.98 ± 0.42	1.69 ± 0.45	2.09 ± 0.42	4.85 ± 0.45	0.0153	0.1042	< .0001
Omental fat pad (g)	0.79 ± 0.10	0.38 ± 0.11	0.48 ± 0.10	0.75 ± 0.11	0.7624	0.5021	0.0032
Cecal digesta dry matter (%)	20.13 ± 0.97	18.57 ± 1.04	17.11 ± 0.97	19.17 ± 1.04	0.2393	0.802	0.0824
Cecal digesta crude protein (%)	4.40 ± 0.27	3.89 ± 0.28	3.40 ± 0.27	4.22 ± 0.28	0.2324	0.5757	0.0233

^1^n = 7-8/group, values are means ± SEM.

^2^Determined using dual-energy X-ray absorptiometry (DXA).

^3^HOMA-IR = homeostasis model assessment-insulin resistance; LDL = low-density lipoprotein; HDL = high-density lipoprotein; NEFA = non-esterified fatty acids; HCR = high capacity runner rats; LCR = low capacity runner rats; EX = voluntary exercise; SED = sedentary.

### Cecal Microbiota

A total of 270,237 reads were obtained, with an average of 9,007 reads (range = 4,063–15,164) per sample. The dataset was rarified to 4,063 OTU for analysis of diversity and species richness. Alpha diversity measures suggested tendencies for differences in phylogenetic diversity due to strain, with LCR rats tended to have greater gut microbiota diversity than HCR rats [observed species at the 97% level: 271 ± 27 vs. 249 ± 32 (*p* = 0.054); *Chao 1* index: 333 ± 29 vs. 311 ± 36 (*p* = 0.086); phylogenetic diversity whole tree matrix: 22.7 ± 1.6 vs. 21.9 ± 1.8 (*p* = 0.191)]. Exercise, however, did not appear to affect alpha diversity.

**Fig 1:**
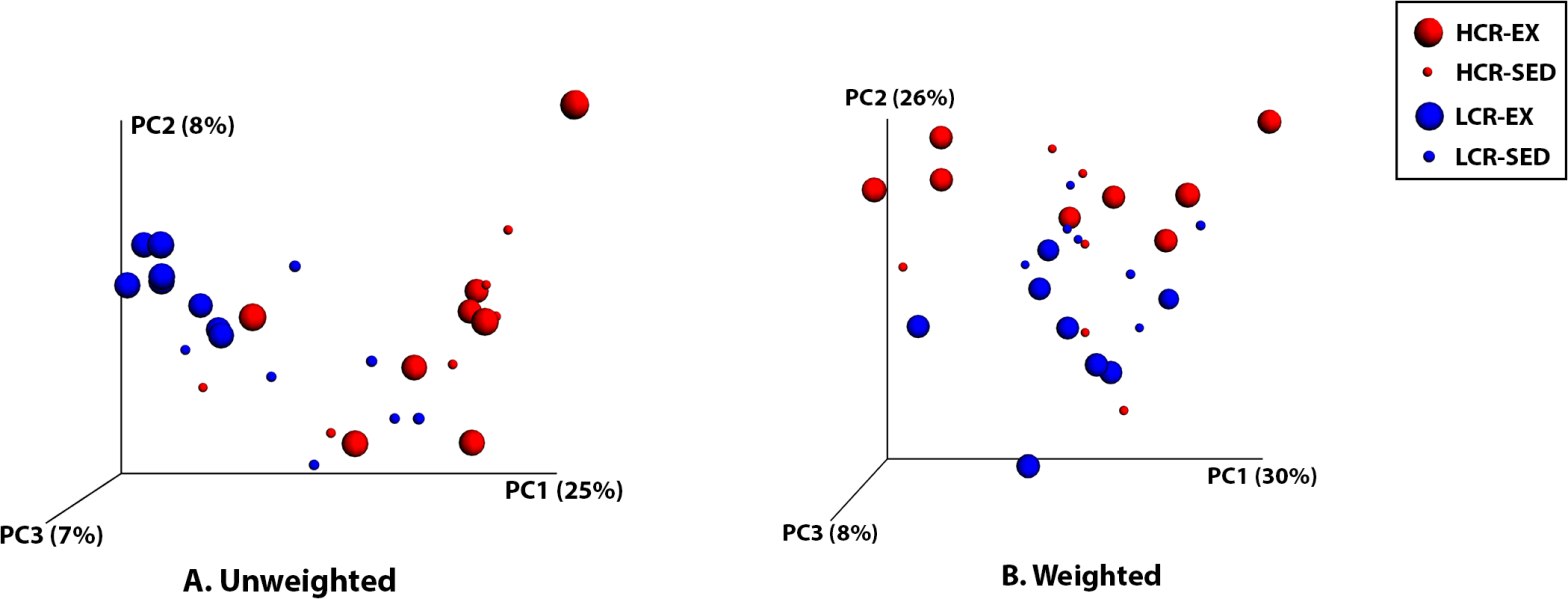
PCoA Plots of Unweighted and Weighted UniFrac Distances. Unweighted **(A)** and weighted **(B)** PCoA was performed based on the UniFrac distance matrix generated from sequencing cecal 16S rRNA genes in samples from HCR and LCR rats. Each dot represents a sample from HCR-EX (big red dots), HCR-SED (small red dots), LCR-EX (big blue dots), and LCR-SED (small blue dots) rats.

Principal coordinate analysis (PCoA) of unweighted UniFrac distances performed on the 97% OTU abundance matrix obtained showed that the beta diversity of gut microbial communities was more similar within LCR or HCR rats (*p* = 0.001 two-tailed two-sample Monte Carlo t-test with 1000 iterations) than between the two lines (Fig 1A). Fig 1A demonstrates that voluntary exercise, shifting the gut microbial communities of LCR rats, clustered them in one area that separated them from the other treatment groups. PCoA of weighted UniFrac distances between samples based on their 97% OTU composition and abundances indicated that gut microbial communities of LCR rats were more similar to each other than to HCR rats (*p* = 0.01) and that exercised rats were more similar to each other than to sedentary rats (p = 0.04) (Fig 1B).

### Taxonomic Shifts due to Exercise

Greengenes classifier assigned usable raw reads to 9 phyla, 39 families, and 66 genera. Although a number of taxa were identified at each NCBI taxonomic hierarchy level, only a few accounted for the majority at each level. At the phyla level, *Bacteroidetes*, *Firmicutes and Proteobacteria* represented approximately 99% of the sequences, while the rest of the 6 phyla represented < 1% of total sequences.

The most abundant phyla included *Firmicutes* (64.8% of sequences), *Bacteroidetes* (20.1% of sequences), *Proteobacteria* (14.0% of sequences), *Actinobacteria* (0.3% of sequences), *Spirochaetes* (0.3% of sequences), and Deferribacteres (0.2% of sequences). Eleven wk of voluntary wheel running and HFD feeding led to significant line by treatment interactions in gut microbial community structure (*p*<0.05, Table 3), including shifts in abundances of *Firmicutes*, *Proteobacteria* and *Cyanobacteria* at the phylum level. Exercise decreased (*p*<0.05) the relative abundance of *Firmicutes* in LCR rats, but increased (*p*<0.05) their relative abundance in HCR rats. In contrast, the relative abundances of *Proteobacteia* and *Cyanobacteria* were increased (*p*<0.05) with exercise in LCR rats, but decreased (*p*<0.05) in HCR rats. Relative abundance of *Bacteroidetes* and the *Bacteroidetes*:*Firmicutes* ratio did not differ among groups.

**Table 3:** Bacterial phyla, families, and genera of cecal digesta in rats after 11 wk of diet and voluntary wheel running interventions^1^.

			HCR^2^		LCR		*p* values		
Phylum	Family	Genus	EX	SED	EX	SED	Line	Trt	Line*Trt
			% of sequence						
Firmicutes			70.74 ± 4.55	61.46 ± 4.87	55.46 ± 4.55	72.82 ± 4.87	0.6199	0.4706	0.0120
	*Clostridiales* ^*3*^	*undefined*	*22*.*75* ± *2*.*36*	*22*.*65* ± *2*.*52*	*23*.*91* ± *2*.*36*	*29*.*16* ± *2*.*52*	0.1291	0.3023	0.2838
	*Lachnospiraceae*		*21*.*44* ± *2*.*79*	*15*.*92* ± *2*.*99*	*6*.*97* ± *2*.*79*	*12*.*33* ± *2*.*99*	0.0044	0.9796	0.0713
		*Blautia*	10.14 ± 2.20	5.60 ± 2.35	0.24 ± 2.20	3.09 ± 2.35	0.0114	0.7135	0.1169
		*Coprococcus*	3.03 ± 0.70	3.85 ± 0.74	3.50 ± 0.70	2.47 ± 0.74	0.5357	0.8882	0.2081
		*Dorea*	2.17 ± 0.51	1.13 ± 0.55	0.29 ± 0.51	0.83 ± 0.55	0.0511	0.6423	0.1497
		*Ruminococcus*	1.23 ± 0.27	1.35 ± 0.29	0.57 ± 0.27	1.24 ± 0.29	0.1792	0.1760	0.3353
		*Roseburia*	0.10 ± 0.04	0.13 ± 0.04	0.04 ± 0.04	0.05 ± 0.04	0.1048	0.6044	0.8603
		*undefined*	4.77 ± 1.00	3.86 ± 1.07	2.32 ± 1.00	4.65 ± 1.07	0.4326	0.4994	0.1329
	*Ruminococcaceae*		16.01 ± 2.32	13.61 ± 2.48	14.27 ± 2.32	23.52 ± 2.48	0.1009	0.1655	0.0227
		*Ruminococcus*	4.91 ± 1.49	2.44 ± 1.60	4.24 ± 1.49	9.40 ± 1.60	0.0520	0.3921	0.0205
		*Oscillospira*	3.02 ± 0.80	4.12 ± 0.85	6.31 ± 0.80	4.39 ± 0.85	0.0406	0.6255	0.0797
		*undefined*	8.06 ± 1.46	7.06 ± 1.56	3.72 ± 1.46	9.72 ± 1.56	0.5807	0.1104	0.0277
	*Erysipelotrichaceae*		*4*.*69* ± *1*.*68*	*5*.*18* ± *1*.*80*	*3*.*24* ± *1*.*68*	*0*.*90* ± *1*.*80*	0.1172	0.5837	0.4091
		*Coprobacillus*	2.16 ± 0.65	1.47 ± 0.70	0.00 ± 0.65	0.26 ± 0.70	0.0196	0.7515	0.4895
		*Eubacterium*	0.41 ± 0.17	0.24 ± 0.18	0.00 ± 0.17	0.05 ± 0.18	0.1044	0.7415	0.5456
		*Allobaculum*	1.56 ± 1.52	3.24 ± 1.63	3.30 ± 1.52	0.45 ± 1.63	0.7423	0.7139	0.1625
		*undefined*	0.49 ± 0.13	0.18 ± 0.14	0.01 ± 0.13	0.12 ± 0.14	0.0552	0.4497	0.1184
	*Veillonellaceae*		1.16 ± 0.19	0.95 ± 0.21	0.47 ± 0.19	0.94 ± 0.21	0.0888	0.5107	0.0939
		*Phascolarctobacterium*	1.09 ± 0.22	0.74 ± 0.24	0.14 ± 0.22	0.77 ± 0.24	0.0562	0.5480	0.0413
		*undefined*	0.06 ± 0.10	0.19 ± 0.10	0.32 ± 0.10	0.15 ± 0.10	0.2747	0.8183	0.1457
	*Peptococcaceae*		1.12 ± 0.16	0.78 ± 0.17	0.32 ± 0.16	0.58 ± 0.17	0.0057	0.8226	0.0861
		*rc4-4*	0.94 ± 0.16	0.67 ± 0.17	0.16 ± 0.16	0.43 ± 0.17	0.0057	0.9974	0.1157
		*undefined*	0.18 ± 0.04	0.12 ± 0.04	0.16 ± 0.04	0.14 ± 0.04	0.9510	0.3721	0.5669
	*Peptostreptococcaceae*	*undefined*	*0*.*74* ± *0*.*55*	*0*.*11* ± *0*.*58*	*2*.*05* ± *0*.*55*	*0*.*60* ± *0*.*58*	0.1199	0.0756	0.4710
	*Streptococcaceae*		*0*.*96* ± *0*.*58*	*1*.*26* ± *0*.*62*	*0*.*10* ± *0*.*58*	*1*.*90* ± *0*.*62*	0.8530	0.0934	0.2244
		*Streptococcus*	0.74 ± 0.38	0.42 ± 0.40	0.07 ± 0.38	1.76 ± 0.40	0.3978	0.0901	0.0165
		*Lactococcus*	0.22 ± 0.36	0.84 ± 0.39	0.03 ± 0.36	0.13 ± 0.39	0.2397	0.3422	0.5045
	*Clostridiaceae*		*0*.*79* ± *0*.*46*	*0*.*17* ± *0*.*49*	*2*.*57* ± *0*.*46*	*0*.*66* ± *0*.*49*	0.0246	0.0133	0.1874
		*SMB53*	0.15 ± 0.09	0.03 ± 0.10	0.32 ± 0.09	0.14 ± 0.10	0.1392	0.1111	0.7424
		*Clostridium*	0.07 ± 0.03	0.04 ± 0.03	0.15 ± 0.03	0.03 ± 0.03	0.3179	0.0351	0.1480
		*undefined*	0.56 ± 0.37	0.10 ± 0.40	2.00 ± 0.37	0.48 ± 0.40	0.0262	0.0165	0.1819
	*Christensenellaceae*	*undefined*	*0*.*34 ± 0*.*04*	*0*.*26 ± 0*.*05*	*0*.*13 ± 0*.*04*	*0*.*25 ± 0*.*05*	0.0260	0.5859	0.0297
	*Turicibacteraceae*	*Turicibacter*	0.32 ± 0.17	0.02 ± 0.18	0.10 ± 0.17	0.03 ± 0.18	0.5244	0.2957	0.5110
	*Lactobacillaceae*	*Lactobacillus*	*0*.*16 ± 0*.*47*	*0*.*37 ± 0*.*50*	*1*.*31 ± 0*.*47*	*0*.*75 ± 0*.*50*	0.1278	0.7169	0.4416
Bacteroidetes			22.89 ± 3.18	21.18 ± 3.40	18.85 ± 3.18	17.29 ± 3.40	0.2393	0.6238	0.9828
	*Bacteroidaceae*	*Bacteroides*	*9*.*02 ± 1*.*58*	*6*.*91 ± 1*.*68*	*4*.*53 ± 1*.*58*	*6*.*07 ± 1*.*68*	0.1149	0.8620	0.2729
	*S24-7*	*undefined*	*5*.*84 ± 0*.*97*	*5*.*78 ± 1*.*04*	*6*.*77 ± 0*.*97*	*5*.*73 ± 1*.*04*	0.6649	0.5881	0.6302
	*Paraprevotellaceae*		4.66 ± 1.67	3.75 ± 1.79	4.85 ± 1.67	2.29 ± 1.79	0.7141	0.3252	0.6382
		*Prevotella*	4.64 ± 1.44	2.48 ± 1.54	4.73 ± 1.44	2.27 ± 1.54	0.9936	0.1239	0.8924
		*CF231*	0.02 ± 0.01	0.05 ± 0.01	0.01 ± 0.01	0.02 ± 0.01	0.1138	0.0541	0.4752
	*Prevotellaceae*	*Prevotella*	*1*.*75 ± 0*.*71*	*2*.*86 ± 0*.*76*	*1*.*40 ± 0*.*71*	*2*.*24 ± 0*.*76*	0.5116	0.1982	0.8544
	*Porphyromonadaceae*	*Parabacteroides*	*0*.*77 ± 0*.*16*	*0*.*56 ± 0*.*17*	*0*.*29 ± 0*.*16*	*0*.*26 ± 0*.*17*	0.0222	0.4711	0.5963
	*Bacteroidales* ^*4*^	*undefined*	*0*.*31 ± 0*.*15*	*0*.*51 ± 0*.*17*	*0*.*38 ± 0*.*15*	*0*.*19 ± 0*.*17*	0.4596	0.9889	0.2271
	*Odoribacteraceae*	*Butyricimonas*	0.29 ± 0.09	0.40 ± 0.10	0.23 ± 0.09	0.21 ± 0.10	0.1946	0.6537	0.5117
	*Rikenellaceae*		*0*.*24 ± 0*.*11*	*0*.*41 ± 0*.*12*	*0*.*40 ± 0*.*11*	*0*.*30 ± 0*.*12*	0.8152	0.7686	0.2658
		*undefined*	0.23 ± 0.11	0.40 ± 0.12	0.40 ± 0.11	0.30 ± 0.12	0.7895	0.7824	0.2416
Proteobacteria			5.03 ± 3.61	16.23 ± 3.85	24.35 ± 3.61	10.09 ± 3.85	0.0891	0.6852	0.0021
	*Helicobacteraceae*		*3*.*22 ± 1*.*96*	*6*.*02 ± 2*.*09*	*12*.*11 ± 1*.*96*	*5*.*51 ± 2*.*09*	0.0491	0.3569	0.0284
		*Helicobacter*	0.07 ± 0.05	0.29 ± 0.05	0.03 ± 0.05	0.04 ± 0.05	0.0050	0.0238	0.0386
		*Flexispira*	0.70 ± 0.25	0.66 ± 0.27	0.00 ± 0.25	0.00 ± 0.27	0.0150	0.9374	0.9374
		*undefined*	2.45 ± 2.01	5.07 ± 2.12	12.08 ± 2.01	5.47 ± 2.12	0.0233	0.3459	0.0352
	*Desulfovibrionaceae*		*1*.*74 ± 2*.*97*	*9*.*96 ± 3*.*17*	*12*.*24 ± 2*.*97*	*4*.*55 ± 3*.*17*	0.4161	0.9318	0.0155
		*Desulfovibrio*	0.30 ± 0.15	0.39 ± 0.16	0.27 ± 0.15	0.72 ± 0.16	0.3448	0.0996	0.2568
		*undefined*	1.44 ± 2.94	9.57 ± 3.14	11.96 ± 2.94	3.83 ± 3.14	0.4394	1.0000	0.0128
	*Alcaligenaceae*	*Sutterella*	*0*.*03 ± 0*.*07*	*0*.*21 ± 0*.*07*	*0*.*00 ± 0*.*07*	*0*.*01 ± 0*.*07*	0.1494	0.1955	0.2510
Deferribacteres	Deferribacteraceae	*Mucispirillum*	0.42 ± 0.18	0.12 ± 0.19	0.07 ± 0.18	0.03 ± 0.19	0.2421	0.3587	0.4747
Spirochaetes	*Spirochaetaceae*	*Treponema*	0.40 ± 0.11	0.22 ± 0.12	0.31 ± 0.11	0.26 ± 0.12	0.8234	0.3235	0.5663
Actinobacteria			0.38 ± 0.09	0.26 ± 0.10	0.36 ± 0.09	0.24 ± 0.10	0.8356	0.2199	0.9557
	*Coriobacteriaceae*		*0*.*36 ± 0*.*06*	*0*.*22 ± 0*.*06*	*0*.*17 ± 0*.*06*	*0*.*22 ± 0*.*06*	0.1253	0.4439	0.1234
		*Adlercreutzia*	0.30 ± 0.04	0.17 ± 0.05	0.09 ± 0.04	0.09 ± 0.05	0.0029	0.1585	0.1072
		*undefined*	0.06 ± 0.03	0.06 ± 0.03	0.09 ± 0.03	0.13 ± 0.03	0.1105	0.5726	0.4608
Tenericutes			0.07 ± 0.11	0.38 ± 0.12	0.04 ± 0.11	0.19 ± 0.12	0.3795	0.0650	0.4949
	*Mycoplasmataceae*	*Mycoplasma*	*0*.*00 ± 0*.*11*	*0*.*33 ± 0*.*11*	*0*.*00 ± 0*.*11*	*0*.*00 ± 0*.*11*	0.1481	0.1481	0.1481
	*RF39* ^*5*^	*undefined*	*0*.*07 ± 0*.*04*	*0*.*04 ± 0*.*04*	*0*.*04 ± 0*.*04*	*0*.*19 ± 0*.*04*	0.1671	0.1586	0.0581
Cyanobacteria	YS2^6^	*undefined*	0.05 ± 0.08	0.15 ± 0.09	0.35 ± 0.08	0.07 ± 0.09	0.2091	0.2915	0.0421

^1^n = 7-8/group, values are means ± SEM.

^2^HCR = high capacity runner rats; LCR = low capacity runner rats; EX = voluntary exercise; SED = sedentary.

^3^Unknown family within order *Clostridiales*.

^4^Unknown family within order *Bacteroidales*.

^5^Unknown family within order *RF39*.

^6^Unknown family within order *YS2*.

In our experimental animal model of human menopause (i.e., OVX+HFD), 11 wk of voluntary wheel running and HFD feeding led to significant shifts (*p*<0.05) in the relative abundances of several cecal bacterial families, including *Lachnospiraceae*, *Ruminococcaceae*, *Peptococcaceae*, *Clostridiaceae*, *Christensenellaceae*, *Porphyromonadaceae*, *Helicobacteraceae*, *Desulfovibrionaceae*, and an undefined family within the order YS2 of the *Cyanobacteria* phylum (Table 3). Significant line by treatment interactions (*p*<0.05) were observed in the relative abundances of *Ruminococcaceae*, *Christensenellaceae*, *Helicobacteraceae*, *Desulfovibrionaceae*, and an undefined family in the *Cyanobacteria* meaning habitual exercise differentially affected these populations depending on intrinsic aerobic fitness (i.e., HCR line vs. LCR line). Exercise decreased the relative abundance of *Ruminococcaceae* in LCR rats, but increased in HCR rats, with the *Ruminococcus* and an undefined genus showing similar shifts at the genus level. Similarly, exercise decreased the relative abundance of *Christensenellaceae* in LCR rats, but increased in HCR rats. Conversely, the relative abundances of *Helicobacteraceae*, *Desulfovibrionaceae* and an undefined family in the *Cyanobacteria* phylum increased with exercise in LCR rats, but decreased in HCR rats. Interestingly, sedentary HCR rats had higher (*p*<0.05) *Helicobacter* than sedentary LCR rats, but exercise reduced the abundance of *Helicobacter* in HCR rats.

Line main effects were also demonstrated, with HCR rats having greater (*p*<0.05) relative abundances of *Lachnospiraceae*, *Peptococcaceae*, and *Porphyromonadaceae* than LCR rats (Table 3). Main effects of line and exercise were observed in the *Clostridiaceae* family, with HCR rats having lower (*p*<0.05) relative abundance of *Clostridiaceae* compared to LCR rats. Exercise increased (*p*<0.05) the relative abundances of *Clostridiaceae* and *Clostridium* in the cecal digesta of these rats (Table 3).

### Correlation of Microbial Abundance and Physiological Profile

**Fig 2:**
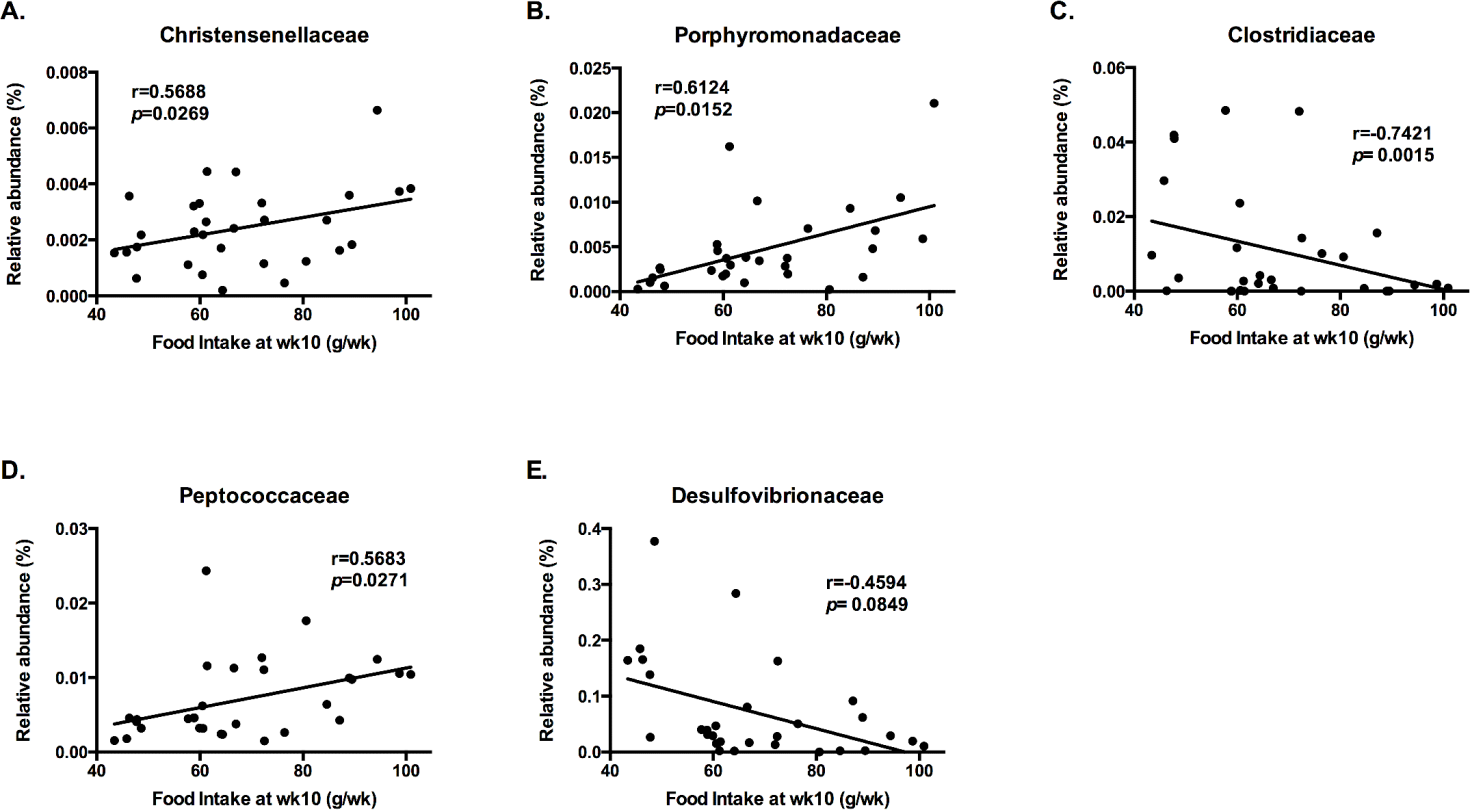
Relative Microbial Abundance and Food Intakes Correlation. Correlations between the relative abundance of the bacterial communities (OTU) and food intake at wk11 of dietary and exercise interventions. Pearson correlation coefficients (r) are shown for each taxon: (A) Christensenellaceae, (B) Porphyromonadaceae, (C) Clostridiaceae, (D) Peptococcaceae, and (E) Desulfovibrionaceae, with the associated *p* values.

**Fig 3:**
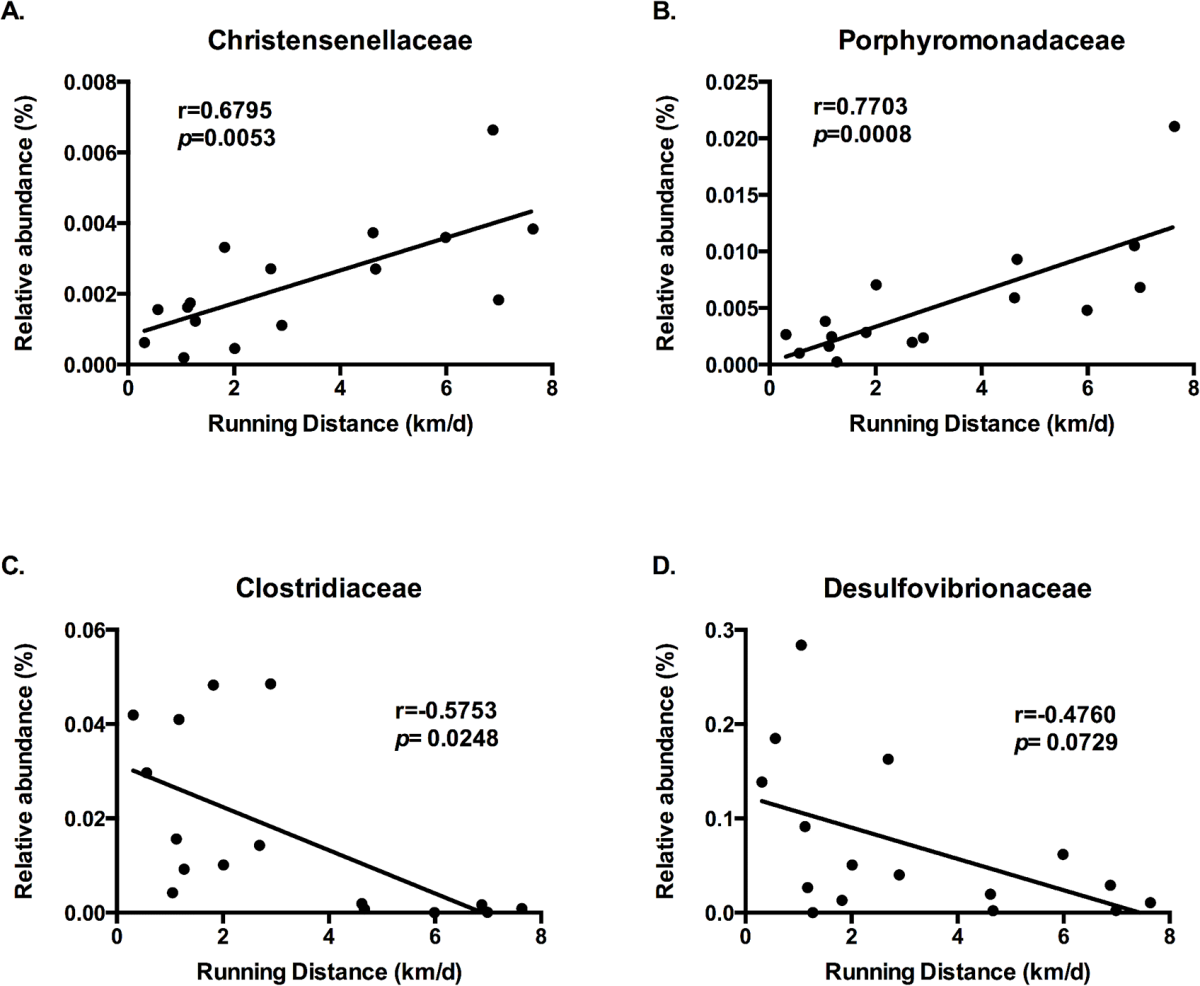
Relative Microbial Abundance and Running Distance Correlation. Correlations between the relative abundance of the bacterial communities (OTU) and running distance (km/d). Pearson correlation coefficients (r) are shown for each taxon: (A) Christensenellaceae, (B) Porphyromonadaceae, (C) Clostridiaceae, and (D) Desulfovibrionaceae, with the associated *p* values.

Correlations between relative microbial abundance and certain physiological parameters were assessed, and several microbial families were found to be significantly correlated with BW change, food intake, and running distance. Relative abundance of *Christensenellaceae* (Fig 2A), *Porphyromonadaceae* (Fig 2B), and *Peptococcaceae* (Fig 2D) were positively correlated with food intake at wk 11 of the dietary and voluntary exercise intervention (Fig 2), whereas *Clostridiaceae* (Fig 2C) and *Desulfovibrionaceae* were negatively correlated with food intake (Fig 2E). *Christensenellaceae* (Fig 3A) and *Porphyromonadaceae* (Fig 3B) were positively correlated, while *Clostridiaceae* (Fig 3C) and *Desulfovibrionaceae* were negatively correlated with running distance (Fig 3D). No significant correlation was found between relative microbial abundances and percent body fat and percent lean body mass.

## Discussion

This study utilized a unique model of human menopause, the HFD-fed OVX rat, by using rats selectively bred for either high or low intrinsic aerobic fitness (i.e., HCR and LCR, respectively) to determine how habitual wheel running exercise affects gut microbial communities. The most prominent findings from this study were that (1) voluntary exercise significantly lowered the body and fat mass, and normalized fasting NEFA concentrations of LCR rats to match that of HCR-SED rats, despite running only one third the distance compared to HCR rats, (2) voluntary exercise shifted the gut microbial communities of LCR rats and separated them from other treatment groups, (3) higher food intake and running distance were associated with higher relative abundance of *Christensenellaceae*, regardless of rat lines, and (4) higher food intake and running distance were associated with lower relative abundance of *Clostridiaceae* and *Desulfovibrionaceae*.

The HFD + OVX intervention resulted in significant weight gain in both LCR and HCR rats (HCR-SED: 304.9 ± 9.6 g, LCR-SED: 360.7± 9.6 g) when compared to similarly-aged female, sham-operated rats fed normal rodent chow in a previous study using this model (HCR: 219.98 ± 3.83 g, LCR: 252.27 ± 3.40 g) [27] and compared to younger intact LCR rats fed HFD rats reported by Naples and colleagues [28]. Likely, the greater BWs in the current study were attributed to the impact of OVX, which is known to increase BW in rodents and humans [29, 30]. Our research group had previously examined body composition of intact vs. OVX-HCR and-LCR rats. LCR responded to OVX with a significantly greater percent body fat compare to intact HCR, LCR, and OVX-HCR rats [10]. OVX-HCR and-LCR rats in this study exhibited similar percent body fat when compare to our previous study. Surprisingly, 11-wk of voluntary exercise lowered percent body fat of OVX-LCR rats to that identical to intact female LCR rats [10].

The distances run, as assessed through voluntary wheel running, in the current study were shorter than a previous study assessing voluntary wheel running distance of younger intact female HCR/LCR rats without HFD feeding [27]; indeed OVX is known to reduce physical activity in rodents and humans as well [29, 31]. Despite the shorter running distance in the current study, voluntary wheel running significantly lowered the body and fat mass, and normalized fasting NEFA concentrations of LCR rats to match that of HCR-SED rats. These dramatic reductions happened to LCR rats while running only one third of distance compare to HCR rats. The effect of wheel running on BW in the current study of OVX rats fed HFD was however somewhat paradoxical, with running reducing BW gain (as expected) in LCR but increasing BW in HCR (not expected). In a previous study of rats fed normal chow diet, wheel running did not affect BW [27]. The weight gain of HCR-EX rats in the current study likely resulted from overconsumption due to a combination of higher energy density of HFD and overcompensation for the increased energy expenditure imparted by the high volume of exercise maintained in this group. Notably, exercise did not affect food intake of LCR rats, suggesting that the beneficial metabolic changes observed in LCR-EX rats might be due to lower energy harvest by the host (e.g., reduced digestive and/or absorptive capacities) or via gut microbiota. Gut microbial communities could potentially be altered due to increased core and intestinal temperature [32], increased gut transit rate (which may lead to altered substrate load or macronutrient composition that reaches the hind gut), and compromised intestine blood flow occurred during exercise [33]. Specific mechanisms responsible for this unexpected finding need to be further investigated.

As expected, line effects were observed for physiologic outcomes, including adiposity and blood lipid and insulin concentrations such that high-fit (HCR) rats, for the most part, had more favorable metabolic profiles compared to low-fit (LCR) rats. Exercise normalized fasting NEFA of LCR rats in the current study; however, despite the significant reduction in BW and fat mass in LCR rats, exercise did not affect blood cholesterol concentrations. Paradoxically, exercise elevated fasting NEFA concentrations in HCR rats to those similar to LCR-SED rats. These findings suggest that voluntary wheel running may benefit LCR rats more than HCR rats in this setting of HFD+OVX, despite the remarkable running distance observed in HCR rats.

This unique rat model that was selectively bred for high- and low- intrinsic aerobic capacity has been extensively studied in regards to the distinct physiological and metabolic differences. To our knowledge, this report provides the first investigation of the gut microbiota of these animals. Recent studies have linked gut microbiota with the development of obesity, metabolic syndrome, and associated comorbidities [4, 34, 35], yet the connections between gut microbiota and menopause induced obesity remain unclear. Furthermore, the beneficial effects of physical activity on promoting weight loss and metabolism might be, in part, mediated by shifts in gut microbiota and/or their metabolic activity. Herein, we demonstrated that both intrinsic fitness (i.e. rat lines) and physical activity affected the composition of cecal microbiota in an animal model of human menopause. Voluntary wheel running appeared to shift gut microbiota of LCR rats away from the other groups, but this did not occur in HCR rats. Assessment of gut microbiota communities in this study did not support our hypothesis that increased physical activity would shift the microbiota of LCR rats, resulting in populations more similar to that of HCR rats. Similar to what we had observed in the improvement of metabolic health, voluntary wheel running appears to impact the gut microbiota of LCR rats to a greater extent compared to HCR rats. With the distinct genetic differences between HCR and LCR rats, potential host genome-gut microbial interactions might contribute to the differential impacts on metabolic changes between two rat lines.

HCR rats tended to have lower bacterial alpha-diversity compared to LCR rats. This result is in disagreement with a previously published dataset reporting a reduction in alpha-diversity of obese vs. lean twins [1]. However, it agrees with previous research assessing gut microbiota in adult humans with or without type 2 diabetes [36]. In that study, diabetic subjects with BMI>31 had higher fecal bacterial alpha-diversity than the lean diabetic subjects and the control group. Exercise has been shown to increase the richness of gut bacterial diversity. Daily treadmill training (30 min of exercise, 5 days per wk) for 4 wk increased fecal bacterial alpha-diversity in healthy Wistar rats, obese Zucker rats and spontaneous hypertensive Wistar-Kyoto rats [37]. Similarly, voluntary wheel running for 12 wk increased fecal bacterial alpha-diversity in C57BL/6 mice fed a 60% kcal fat HFD [38]. A recently published human study showed that gut microbiota diversity of professional athletes was significantly higher than their size and age/gender-matched controls [39]. Eleven wk of voluntary wheel running in the current study, however, did not affect cecal bacterial alpha-diversity in HFD-fed OVX HCR or LCR rats. Whether the lack of effect here was due to lack of ovarian function, exposure to HFD, or selective breeding per se cannot be determined and speaks to the complexity of the interrelationships between genetics, behavior and hormonal changes. Clearly, many questions remain unanswered and deserve further investigation.

Increased relative abundance of *Firmicutes* and decreased relative abundance of *Proteobacteria* were observed in HCR rats with voluntary wheel running in the current study. Similar shifts were reported in a recent treadmill training study in healthy Wistar rats, obese Zucker rats and spontaneous hypertensive Wistar-Kyoto rats [37]. In contrast, we observed that LCR rats had decreased *Firmicutes* abundance and increased *Proteobacteria* with voluntary wheel running. A similar reduction of *Firmicutes* abundance has been reported with voluntary wheel running in mice [38]. Relative abundance of *Bacteroidetes* in the current study did not differ between lines or with voluntary exercise, conflicting with the results of previous studies. Evans et al. [38] reported that voluntary wheel running increased *Bacteroidetes* in mice fed low- and high-fat diets, while Petriz et al. [37] demonstrated that *Bacteroidetes* was reduced with treadmill training in Wistar rats, but not obese Zucker rats. In humans, professional athletes have been reported to have lower *Bacteroidetes* abundance compared to controls [39]. These conflicting results suggest that host species, location and origin of animals, and type of exercise may affect host-microbial interactions and microbial populations differently.

The *Christensenellaceae* family was recently found to be a highly heritable taxa of the human microbiota, and a high abundance of this family was associated with low BMI [40]. Here we found significantly higher relative abundance of *Christensenellaceae* abundance in HCR compared to LCR rats, suggesting the potential association of this family with body mass. We also found that the relative abundance of *Christensenellaceae* has a markedly positive correlation with food intake, BW change and running distance. However, LCR rats had significantly lower body mass with wheel running intervention, but their relative abundance of *Christensenellaceae* had decreased.

Exercise is known to result in gastrointestinal tract distress in humans, with plausible causes including accelerated gut transit of digesta [41], increased intraluminal gas [42], and increased intra-abdominal pressure and organ bouncing [43] associated with exercise bouts. Changes of gut transit time may affect nutrient digestion and/or absorption in the small intestine of the host, affecting substrate availability and utilization by gut microbiota, and the growth of bacterial taxa. In this study, relative abundance of nitrate-reducing *Helicobacteraceae* and sulfate-reducing *Desulfovibrionaceae* were increased with exercise in LCR rats, but decreased in HCR rats. *Helicobacter* and undefined genera in the *Helicobacteraceae* and *Desulfovibrionaceae* families had similar changes. Because increased organic sulfur-containing substrates (e.g., proteins and mucins) may lead to increased growth of Proteobacteria and sulfate-reducing bacteria, we speculated that the elevated food intake and the potential shorter gut transit time in LCR-EX rats may have altered protein digestibility and amount entering the cecum. Therefore, we measured the dry matter and crude protein concentrations of cecal digesta to test whether more proteinaceous substrates were present in LCR-EX rats. Cecal digesta dry matter was not altered by line or exercise. Although a line by treatment interaction was observed for cecal crude protein concentration, the differences were quite small and not in line with changes in bacteria.

In addition to dietary protein, sulfate-reducing bacteria may also use mucins as substrates because they are high in the sulfur-containing amino acid cysteine [44]. Mucins are the protective layer consisting of glycoprotein that help forming the mucosal barrier lining of gastrointestinal tract, which has recently recognized to play an important role on interacting with gut microbiota and may alter the microbial community composition [5]. High-intensity exercise redirects blood flow to the active tissues such as skeletal muscle and heart [33]. Reduced splanchnic blood flow during exercise is thought to contribute to gut mucosal ischemia, oxidative stress, and increased mucosa permeability due to disruption of intestinal epithelial cell tight junction proteins [45]. Exercise can potentially affect mucus thickness, which may lead to increased abundances of certain bacterial communities. However, further research is needed to investigate this hypothesis.

Our study showed that 11 wk of voluntary wheel running significantly altered the relative abundance of several taxa at the family and genus levels, but that shifts were dependent on rat line (i.e., HCR vs. LCR). The mechanisms of exercise-mediated changes in gut microbiota are unknown, but hormonal and physiological alterations of the host likely play a role. The existence of microbiome-gut-brain axis has been proposed recently, suggesting a link between gut microbiome and behavioral changes. Corticosterone, one of the hormones that release from the adrenal glands when encountering stress, was shown to increase with the ingestion of *Lactobacillus rhamnosus* [20]. Plasma corticosterone has been reported to be higher in germ-free mice compared to SPF mice [46]. Voluntary wheel running has been previously shown to elevate plasma corticosterone in LCR rats compared to HCR rats [27], suggesting that exercise might cause more stress to LCR rats compared to HCR rats. Even though it was not possible to test the mechanism in this study, the observed strain-related differences in cecal microbiota shift are certainly interesting.

It is important to note that no control group was maintained with the development of these two selected lines. The differences observed in the current study were between the divergent rat lines (i.e., HCR and LCR), but not to a wild-type control group. Another limitation is that HCR ran considerably longer distances than LCR; thus, what cannot be determined with certainty is whether the divergent effects with exercise training were indeed due to intrinsic fitness or the volume of exercise. However, controlling the volume of exercise bout would likely have increased stress of LCR rats if forced to exercise beyond their ability. Other exercise-related metabolic changes of the host, such as altered digestion and absorption of nutrients or transit time, compromised intestinal blood flow, altered mucus production, elevated core temperature and increased microbial activity, all of which may increase substrate load in hindgut and further affect gut microbial communities, may have contributed but cannot be verified in this study. Many limitations inevitably exist when studying gut microbiota due to its complexity itself as well as host-microbial interactions, but our results provide targets of study in the future.

Our study demonstrates that, in this model of postmenopausal obesity, voluntary exercise significantly lowered the body and fat mass, and normalized fasting NEFA concentrations of LCR rats, but significantly increased weight gain, fat mass and fasting NEFA concentrations of HCR rats. Voluntary exercise impacts gut microbial communities, but the effects appear to be dependent on the intrinsic aerobic capacity of the host. The elevated weight gain and fat mass of HCR rats were likely the result of an overconsumption of HFD, and the beneficial metabolic changes of LCR rats were likely due to decreased energy harvest by the host gastrointestinal tract and/or via gut microbiota. More studies are needed to elucidate the mechanisms by which exercise and intrinsic aerobic capacity affect gut microbiota communities.
